# Under the chest pain center mechanism, whether the nursing handover affects the nursing efficiency and the outcomes of patients with STEMI in the emergency department? A retrospective study

**DOI:** 10.1186/s12873-023-00773-2

**Published:** 2023-01-12

**Authors:** Zhenyu Luo, Sihui Liu, Yunying Li, Shuyan Zhong

**Affiliations:** 1 Guanyuan Central Hospital, Guangyuan, Sichuan China; 2 The First Affiliated Hospital of Soochow University, Suzhou, China

**Keywords:** Nursing handover, STEMI, Chest pain center, CPC, Handover, Handoffs, Nursing efficiency

## Abstract

**Background:**

The introduction of chest pain centers (CPC) in China has achieved great success in shortening the duration of nursing operations to significantly improve the treatment and outcomes of patients with ST-segment elevation myocardial infarction (STEMI). The nursing handover period is still considered the high incidence period of adverse events because of the distractibility of nurses’ attention, potential interruption, and unclear responsibilities. Under the CPC mechanism, the nursing efficiency and patients’ outcome, whether affected by the nursing handover, is still a knowledge gap in research. This is also the aim of this study.

**Methods:**

A retrospective study was conducted with data from STEMI patients from a tertiary hospital in the north of Sichuan Province from January 2018 to December 2019 through the Chinese CPC database. Patients are divided into handover and non-handover groups according to the time they presented in the Emergency Department. D2FMC, FMC2FE, FMC2BS, FMC2CBR, FMC2FAD, and D2W were selected to measure nursing efficiency. The occurrence of major adverse cardiovascular events, the highest troponin values within 72 h of hospitalization, and the length of hospitalization were selected to measure the patient outcomes. Continuous variables are summarized as mean ± SD, and t-tests of the data were performed. *P*-values < 0.05 (two-tailed) were considered statistically significant.

**Results:**

A total of 231 cases were enrolled, of which 40 patients (17.3%) were divided into the handover period group, and 191 (82.6%) belonged to the non-handover period group. The results showed that the handover period group took significantly longer on items FMC2BS (*P* < 0.001) and FMC2FAD (*P* < 0.001). Still, there were no significant differences in D2FMC and FMC2FE, and others varied too little to be clinically meaningful, as well as the outcomes of patients.

**Conclusion:**

This study confirms that nursing handover impacts the nursing efficiency of STEMI patients, especially in FMC2BS and FMC2FAD. Hospitals should also reform the nursing handover rules after the construction of CPC and enhance the triage training of nurses to assure nursing efficiency so that CPC can play a better role.

## Background

ST-segment elevation myocardial infarction (STEMI) is a time-sensitive and fatal cardiovascular disease (CVD). Along with an increase in risk factors and an aging population, studies predict that 75 million people will suffer from CVD, causing 39 million deaths between 2016 and 2030. STEMI has attracted more attention in China [1]. To conduct the early reperfusion of myocardial ischemia and achieve a better prognosis for patients [2–5], China started to build up the Chest Pain Center(CPC) in 2002 [2], consisting of several departments. As the department for diagnosis, early treatment, and preoperative preparation, the process of the ED is regarded as a vital part of the whole treatment and is strictly regulated by the CPC.

Nursing handover has always been regarded as an essential part of nursing work, which can transfer the patient’s information and ensure the continuity of the patient’s treatment plan [6]. Still, it is also prone to adverse events [7]. Since numerous studies have proved that the quality of handover is closely related to care quality and patient safety [8], a series of relevant mechanisms have been recommended by experts to assure the quality of handover [9, 10]. However, a study also indicated that the actual situation of the emergency department is different from that of many theories [11], and it is even harmful to follow experts’ recommendations blindly. However, studies show that the introduction of the CPC mechanism has made significant progress in treating patients with STEMI in China [2, 5, 12, 13]. However, several studies have also shown that many factors still contribute to delayed reperfusion in patients under the CPC mechanism [14, 15]. This paper aims to investigate whether nursing handover delays the nursing efficiency of the process in the ED and thus affects patient prognosis under the CPC mechanism.

The CPC mechanism sets many time limits for the nursing operations of patients with STEMI. It requires data to be uploaded to the national chest pain center database in time, providing a data basis for this study [5].

## Methods

### Study design and setting

The study was conducted in a tertiary hospital in mainland China, the largest chest pain center in northern Sichuan, with 1,200 beds, including 80 emergency beds, and treating over 10,000 emergency patients annually. The study was conducted under the Sex, and Gender Equity in Research (SAGER) guidelines and retrospective data was approved by the Ethics Committee of Guangyuan Central Hospital. The requirement for informed consent was waived.

#### Selection of participants

To avoid potential bias caused by the COVID-19 epidemic, patients who attended the tertiary hospital ED from January 2018 to December 2019 were selected as the study population. Patients who met the following criteria were included in the study: 1) age ≥ 18 years; 2) diagnosis of STEMI after ECG; 3) no history of PCI; 4) no anticoagulant use within one month. Exclusion criteria: 1) diagnosis of malignancy; 2) significant lack of information.

#### The setting of the handover period

During the nursing handover. Nurses on duty were asked to check equipment, replenish supplies and clean up 30 min before the official end of their shift. And nurses taking over the shift were asked to start the handover 15 min earlier. They will first hand over the different areas of the department and the ambulances according to their duties, which include: emergency equipment, supplies, and sanitation. This is followed by a handover of the patients present in each area. The handover process uses the SBAR communication tool to hand over the patient’s condition, background, assessment, and recommendations [16]. After confirming that all things and information are corrected, the nurse signs the form to mark the handover as over. There is also a handover (15:30 − 16:15) as the day shift nurses are split into two shifts. According to the hospital’s handover regulation and the actual situation (7:30 − 8:15; 15:30 − 16:15; 17:30 − 18:15; 23:30 − 0:15) was set as handover period.

#### Grouping

Patients were divided into two groups. STEMI patients present during the nursing handover period (7:30 − 8:15; 15:30 − 16:15; 17:30 − 18:15; 23:30 − 0:15) were selected as the handover group (*n* = 40), and STEM patients present in other periods were included in the non-handover group (*n* = 191).

#### Measurements

The following data were extracted from the National Database of Chest Pain Center: demographic characteristics, previous medical history, and patient outcomes (occurrences of major cardiovascular events during hospitalization, highest troponin value within 72 h of hospitalization, length of hospitalization). The data of D2FMC (time from the patient’s arrival at the hospital gate to the first medical contact), FMC2FE(time from the first medical contact to the first ECG), FMC2BS(time from the first medical contact to the blood sampling), BS2CBR(time from the blood sampling to the cardiac biomarkers report), FMC2FAD (time from the cardiac biomarkers report to the first antiplatelet drug)and D2W(time from the patient’s arrival at the hospital gate to the opening of their occluded coronary arteries) were used to measure the nursing efficiency selected based on relative official studies and guidelines [4, 12, 17]. Five of these items are directly related to nursing work, and D2W is also profoundly influenced by nursing work and closely associated with the prognosis of patients.

### Data analysis

Continuous variables are summarized as mean ± SD. Normality testing and following t-tests of the data were performed by SPSS (version 24.0, IBM Corporation). *P*-values < 0.05 (two-tailed) were considered statistically significant.

## Results

A total of 231 cases were enrolled, of which 40 patients (17.3%) were divided into the handover period group, and 191 (82.6%) belonged to the non-handover period group. The subsequent t-test results are shown in Table 1.

The results showed that the handover period group took longer on items: FMC2BS (*P* < 0.001), BS2CBR (*P* = 0.004), FMC2FAD (*P* < 0.001), and D2W (*P* = 0.001). However, except for FMC2BS and FMC2FAD, differences in other items are too small and may not make a difference in clinical practice.

**Table 1:** The baseline characteristics and the results of the t-test

	Handover period group	Non-handover period group	*P*-value
**Baseline characteristics**			
Cases	40(17.3%)	191(82.6%)	
Gender	40	191	0.676
Male	30	137	
Female	10	54	
Age	62.4 (± 2.48)	62.8 (± 4.32)	0.358
BMI	28.92 (± 2.09)	28.75 (± 2.38)	0.684
Current smoking	20 (± 0.50)	104 (± 0.49)	0.610
Dyslipidemia	30 (± 0.43)	155 (± 0.39)	0.378
Diabetes mellitus	18 (± 0.50)	58 (± 0.46)	0.096
Heart failure history	7 (± 0.38)	26 (± 0.34)	0.525
Renal failure history	4 (± 0.30)	23 (± 0.32)	0.716
Previous myocardial infarction	5 (± 0.33)	16 (± 0.27)	0.412
Killip class(arrival)			0.781
I	27	115	
II	6	59	
III	5	12	
IV	2	5	
**Nursing efficiency**			
D2FMC (minutes)	2.92 (± 0.72)	2.8 (± 0.76)	0.589
FMC2FE (minutes)	2.12 (± 0.56)	2.14 (± 0.61)	0.876
FMC2BS (minutes)	9.92 (± 2.60)	7.84 (± 2.36)	0.000**
BS2CBR (minutes)	13. (± 0.64)	12.91 (± 0.77)	0.004*
FMC2FAD (minutes)	8.10 (± 0.95)	6.95 (± 1.14)	0.000**
D2W (minutes)	70.6 (± 2.02)	69.21 (± 2.34)	0.001*
**Outcomes**			
Occurrence of major adverse cardiovascular events	6	29	0.977
Highest troponin values within 72 h of hospitalization(TNT, ng/L)	2542 (± 2206.21)	2252.4 (± 2424.93)	0.486
Hospitalization (days)	13.8 (± 2.13)	13.39 (± 2.45)	0.337

*D2FMC* Time from the patient's arrival at the hospital gate to the first medical contact, *FMC2FE* Time from the first medical contact to the first ECG,  *FMC2BS* Time from the first medical contact to the blood sampling, *BS2CBR* Time from the blood sampling to the cardiac biomarkers report, *FMC2FAD* Time from the first medical contact to the first anti-platelet drug, *D2W* Time from the patient’s arrival at the hospital gate to the opening of their occluded coronary arteries

*p < 0.05; **p < 0.001

## Discussion

For STEMI patients, each hour of D2W delay is correlated with an increase in mortality of approximately 3–8% [3, 18]. Although the care of STEMI patients has progressed substantially since CPC construction was popularized nationally [12], the D2W time can still be accelerated [14, 15]. This study found that although both groups met the requirements of the CPC mechanism, there was a significant delay in the nursing handover group on FMC2BS and FMC2FAD.

To reduce D2W, CPC has set strict time limits for each operation [5], such as FMC2FE < 10 min, FMC2CBR < 20 min, and FMC2FAD < 10 min, most of these operations are carried out by nurses, and all have a common timing starting point: the FMC (first medical contact). Considering the triage role of emergency nurses, the vast majority of STEMI patients have their first medical contact with a nurse [19, 20], after which the ED nurse has many tasks to perform within a short period, such as ECG, blood collection, and assisting the patient with anti-platelet medication. As aging is one of the main risk factors for STEMI [1, 12], blood sampling for STEMI patients could be more complex and time-consuming. In addition, anti-platelet drugs need to be chewed and swallowed so that nurses will instruct and feed water at the bedside.

However, the CPC mechanism fails to regulate a nurse-patient ratio to ensure adequate human resources for the care of STEMI patients. Due to the uneven development of emergency medical services and the lack of human resources for nursing [21, 22]. During the non-nursing handover period, sufficient human resources can enable nurses to carry out multiple operations simultaneously. They can even ask colleagues for help when the process meets troubles (such as blood sampling). Further, a national survey indicates the average nurse-patient ratio is 1:8 during the day, while it can reach 1:23 at night in Chinese general hospitals [21], which will undoubtedly worsen the situation of nursing handover at night. Ensuring adequate human resources is a vital issue to consider when setting up a CPC.

Despite all the competencies required of nurses by the CPC, there is no guidance on the nursing handover, to the extent that many emergency departments with CPCs share a handover regulation with other departments. Only a small number of nurses will stay at the nurse station. In contrast, others have to inspect the whole department and pay attention to hand over a large number of first-aid equipment, drugs, and even environmental sanitation, as well as bedside handover of existing patients. To make matters worse, in many provinces, pre-hospital emergency care is also undertaken by the emergency department, which means that nurses are also required to perform detailed handovers of ambulances [23], which may cost more time and energy. From nursing management, designing a unique set of handover rules for CPCs or upgrading information systems deserves more attention.

As the nursing handover is defined as the handover of nursing responsibilities [24] which is prone to unclear responsibilities, outgoing nurses may be reluctant to take the responsibilities of patients who visit during the handover while incoming nurses are still busy in the handover. This may cause more nurses present in ED during the handover, but fewer nurses to participate in the treatment. At this point, we agree that both the smooth triage of patients and the amicable relationship between the transfer in and out of the nurse can improve the quality of handover, ensure the efficiency of nursing care, and reduce the occurrence of adverse events [25]. This requires that the triage nurses have sufficient knowledge of the disease and triage process, and regular training and assessment are essential. And the working relationship between colleagues considering that there are no managers in the Chinese nursing management system, the leadership of the head nurse will play an important role, which deserves more research.

The physician’s ability to communicate about the condition, the time it takes for the patient to consent to the procedure, and the preparation time in the intervention room may all interfere with D2W, which may account for the lack of significant differences in D2W and patient outcomes. In addition, it was not possible to track ED crowding due to the inherent shortcomings of retrospective studies, which may have influenced it.

### Limitations

Firstly, the sample size was not large enough, this CPC was established in 2018 with few patients in the beginning, and to avoid the interference of the Covid-19 pandemic, only cases from January 2018 to December 2019 were extracted. Secondly, as all data come from the same CPC, conclusions should be drawn cautiously when generalizing and applying. Finally, although we include Killip class, age, etc., as confounding factors to ensure the accuracy of the study results, due to the inherent shortcomings of retrospective studies, this study failed to retract the occurrence of ED crowding or nurse-to-patient ratio, which may also affect the results.

## Conclusion

This study confirms that nursing handover impacts the nursing efficiency of STEMI patients, especially in FMC2BS and FMC2FAD. Hospitals should also reform the nursing handover rules after the construction of CPC and enhance the triage training of nurses to assure nursing efficiency so that CPC can play a better role.

## Data Availability

The datasets generated and analyzed during the current study are not publicly available due protect patient privacy but are available from the corresponding author on reasonable request.

## Abbreviations

ED: Emergency department
CPC: Chest pain center
D2FMC: Time from the patient’s arrival at the hospital gate to the first medical contact
FMC2FE: Time from the first medical contact to the first ECG
FMC2BS: Time from the first medical contact to the blood sampling
BS2CBR: Time from the blood sampling to the cardiac biomarkers report
FMC2FAD: Time from the cardiac biomarkers report to the first antiplatelet drug
D2W: Time from the patient’s arrival at the hospital gate to the opening of their occluded coronary arteries
